# 3β-Chloro-*N*-methoxy-*N*-methyl­cholest-5-ene-24-carboxamide

**DOI:** 10.1107/S160053681204679X

**Published:** 2012-11-28

**Authors:** Karilys González, Karinel Nieves, Abimael D. Rodríguez

**Affiliations:** a Department of Chemistry, University of Puerto Rico, PO Box 23346, San Juan 00931-3346, Puerto Rico

## Abstract

The title compound, C_26_H_42_ClNO_2_, is a 3β-chloro steroid with a Weinreb amide at the C-24 position. The two cyclo­hexane and the cyclo­hexene rings adopt chair and boat conformations, respectively. The cyclo­pentane ring has an envelope conformation.

## Related literature

The title compound was obtained as part of our studies on the synthesis of chlorinated steroids as anti­malarial agents. For chlorination of 3β-hydroxyl-5-Δ steroids, see: Liu *et al.* (2005 ▶). For anti­malarial steroids, see: Corrales *et al.* (2011 ▶); Sharma *et al.* (2008 ▶). For the emerging role of chlorinated lipids and fatty acids in pathology, see: Spickett (2007 ▶). For the use of steryl chlorides as synthetic inter­mediates, see: Ochi *et al.* (1977 ▶). For liquid crystal properties of steryl chlorides, see: Leder (1971 ▶). For chloro­quine-resistant malaria, see: Wellems & Plowe (2001 ▶). For drug resistance in malaria, see: Bloland (2001 ▶).
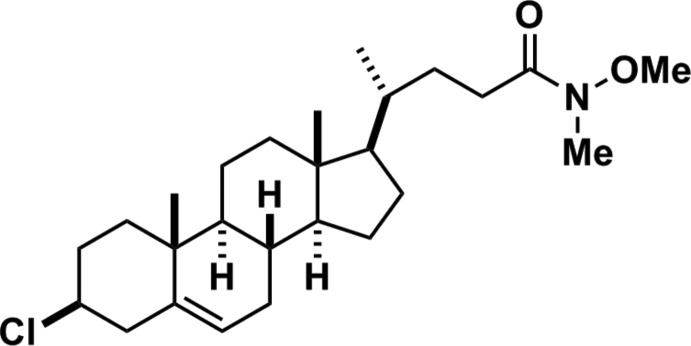


## Experimental

### 

#### Crystal data

C_26_H_42_ClNO_2_
*M* *_r_* = 436.06
Orthorhombic, 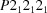

*a* = 7.5263 (2) Å
*b* = 16.2157 (4) Å
*c* = 20.8850 (5) Å
*V* = 2548.89 (11) Å^3^
*Z* = 4
Mo *K*α radiation
μ = 0.17 mm^−1^
*T* = 296 K
0.31 × 0.09 × 0.08 mm

#### Data collection

Bruker APEXII CCD diffractometer
Absorption correction: multi-scan (*SADABS*; Sheldrick, 2008*a* ▶) *T*_min_ = 0.949, *T*_max_ = 0.987
23203 measured reflections
6244 independent reflections
3307 reflections with *I* > 2σ(*I*)
*R*_int_ = 0.035

#### Refinement

*R*[*F* ^2^ > 2σ(*F* ^2^)] = 0.056
*wR*(*F* ^2^) = 0.157
*S* = 1.01
6244 reflections
276 parameters
H-atom parameters constrained
Δρ_max_ = 0.19 e Å^−3^
Δρ_min_ = −0.14 e Å^−3^
Absolute structure: Flack (1983 ▶), 2662 Friedel pairs
Flack parameter: −0.04 (9)

### 

Data collection: *APEX2* (Bruker, 2005 ▶); cell refinement: *SAINT* (Bruker, 1999 ▶); data reduction: *SAINT*; program(s) used to solve structure: *SHELXS97* (Sheldrick, 2008*b* ▶); program(s) used to refine structure: *SHELXL97* (Sheldrick, 2008*b* ▶); molecular graphics: *SHELXTL* (Sheldrick, 2008*b* ▶); software used to prepare material for publication: *SHELXTL*.

## Supplementary Material

Crystal structure: contains datablock(s) I, global. DOI: 10.1107/S160053681204679X/fy2071sup1.cif

Supplementary material file. DOI: 10.1107/S160053681204679X/fy2071Isup2.cdx

Structure factors: contains datablock(s) I. DOI: 10.1107/S160053681204679X/fy2071Isup3.hkl

Additional supplementary materials:  crystallographic information; 3D view; checkCIF report

## Figures and Tables

**Fig. 1.**
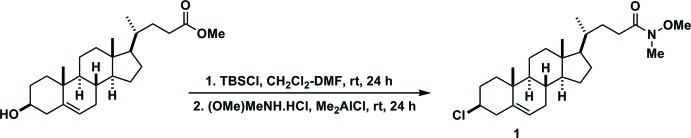
Reaction scheme showing the synthesis of compound 1 from 5-cholenic acid 3β-ol methyl ester with tert-butyldimethyl silyl chloride, N-methoxy N-methyl hydroxylamine hydrochloride, and dimethyl aluminium chloride.

**Fig. 2.**
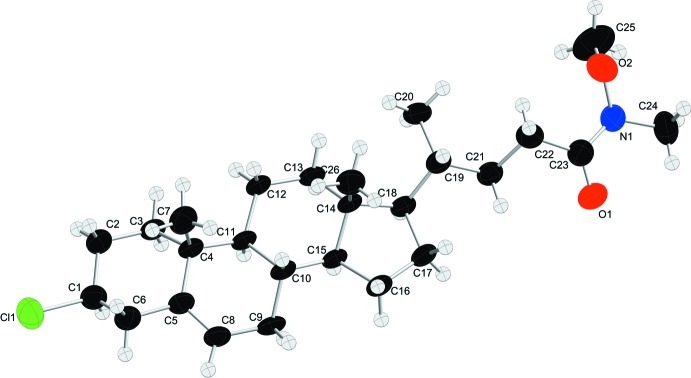
Asymmetric unit of compound 1 with 50% probability displacement ellipsoid for non-hydrogen atoms.

**Fig. 3.**
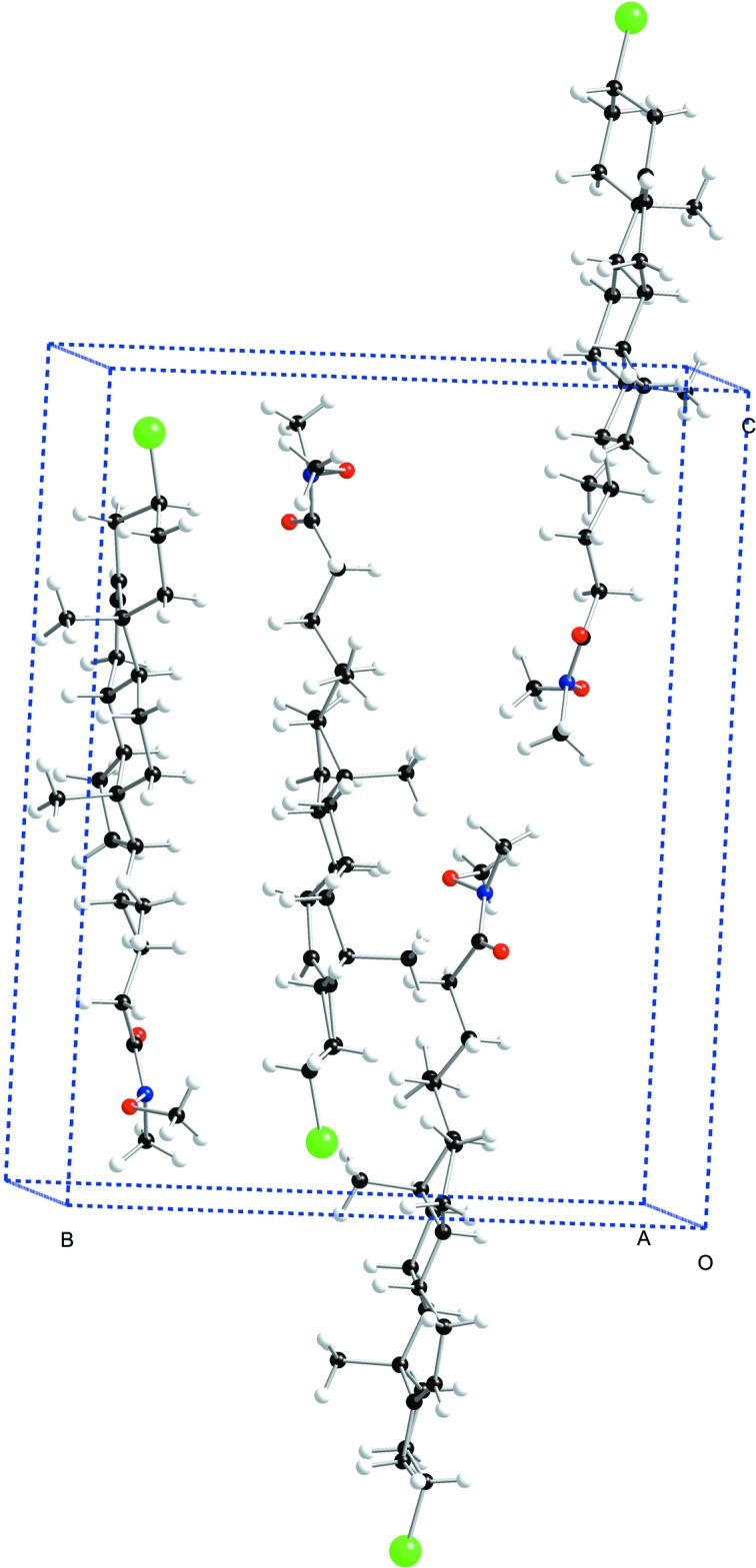
Packing diagram of compound 1.

## supplementary crystallographic information

### Comment

The synthesis of the title compound started with the alcohol protection of 5-cholenic acid 3β-ol methyl ester with *tert*-butyldimethyl silyl chloride. Then it was derivatized to the Weinreb amide in the presence of *N*-methoxy *N*-methyl hydroxylamine hydrochloride and dimethyl aluminium chloride with the concomitant chlorination of the 3β-silyl ether to afford compound 1 with retention of configuration at the C-3 position.

This synthetic route is part of our studies on the synthesis of 3β-chloro steroids as antimalarial agents. There is an urgent need to develop new classes of antimalarial drugs due to the development of resistance by the parasite to the commonly used drugs, such as chloroquine [Bloland (2001) and Wellems and Plowe (2001)]. Compound 1 was screened against *Plasmodium falciparum*, but unfortunely, it showed negligible activity (37.4% inhibition). Our future plans include the synthesis of additional derivatives with different functional groups at C-24. The structure of the title compound was determined by one-dimensional and two-dimensional NMR spectra. To confirm the structure of compound 1, X-ray crystallography was performed.

### Experimental

Compound 1 was synthesized by the reaction of 5-cholenic acid 3β-ol methyl ester (259 mg, 0.67 mmol) and imidazole (114 mg, 1.68 mmol) in 15 ml of CH_2_Cl_2_/DMF (2:1) with 1.0 *M**tert*-butyldimethylsilyl chloride (TBSCl) in CH_2_Cl_2_ (1.0 ml, 1.0 mmol). The reaction mixture was stirred for 24 h at room temperature, quenched with NH_4_Cl _(aq)_ (20 ml), and extracted with CH_2_Cl_2_ (3 *x* 15 ml). The combined organic layers were dried (MgSO_4_) and concentrated *in vacuo*. The crude (378.1 mg) was dissolved in CH_2_Cl_2_ (10 ml), then (OMe)NH(Me).HCl (87.8 mg, 0.9 mmol) and 1.0 *M* Me_2_AlCl in hexane (1.5 ml, 1.5 mmol) were added. The reaction mixture was stirred at room temperature for 24 h, quenched with a solution of 1.0 *M* NaOH_(aq)_, and extracted with CH_2_Cl_2_ (3 *x* 10 ml). The combined organic layers were dried (MgSO_4_) and concentrated *in vacuo*. Purification by flash-Silica Gel column chromatography [Hex/EtOAc (4:1)] afforded the chlorinated steroid 1 as a white solid in 75% yield (217 mg). Crystals of 1 were obtained by slow evaporation from Hex/CH_2_Cl_2_ (2:1) at room temperature. Mp 111–113°C; [α]_D_^20^ -27.0 (*c* 1.0, CHCl_3_); IR (film) ν_max_ 2948, 1662, 1386, 994 cm^-1^; ^1^H NMR (500 MHz, CDCl_3_) δ 5.33 (br d, 1H), 3.71 (m, 1H), 3.65 (s, 3H), 3.13 (s, 3H), 2.53–2.25 (broad envelope, 4H), 2.04–1.71 (broad envelope, 7H), 1.58–0.82 (broad envelope, 14H), 0.98 (s, 3H), 0.91 (d, *J* = 6.5 Hz, 3H), 0.64 (s, 3H); ^13^C NMR (CDCl_3_, 125 MHz) δ 175.1 (C),140.6 (C), 122.3 (CH), 61.1 (CH_3_), 60.1 (CH), 56.5 (CH), 55.8 (CH), 49.9(CH), 43.3 (CH_2_), 42.2 (C), 39.5 (CH_2_), 39.0 (CH_2_), 36.2 (C), 35.4 (CH), 33.2 (CH_2_), 32.1 (CH_3_), 31.7 (CH_2_), 31.6 (CH), 30.6 (CH_2_), 28.7 (CH_3_), 28.0 (CH_2_), 24.1 (CH_2_), 20.8 (CH_2_), 19.1 (CH_3_), 18.4 (CH_3_), 11.7 (CH_3_); HRESIMS *m/z* [*M*+ H]^+^ 436.2977 (calcd for C_26_H_43_NO_2_Cl, 436.2982).

### Refinement

All non-H atoms were refined anisotropically. H atoms were positioned geometrically with C—H=0.96 (CH_3_), 0.97 (CH_2_), 0.98 (CH) Å and constrained with *U*_iso_(H) = 1.5 *U*_eq_(parent) for methyl H and *U*_iso_(H) = 1.2 *U*_eq_(parent) for all other atoms.

### Crystal data

**Table:**

C_26_H_42_ClNO_2_	*F*(000) = 952
*M**_r_* = 436.06	*D*_x_ = 1.136 Mg m^−^^3^
Orthorhombic, *P*2_1_2_1_2_1_	Mo *K*α radiation, λ = 0.71073 Å
Hall symbol: P 2ac 2ab	Cell parameters from 5845 reflections
*a* = 7.5263 (2) Å	θ = 2.7–20.9°
*b* = 16.2157 (4) Å	µ = 0.17 mm^−^^1^
*c* = 20.8850 (5) Å	*T* = 296 K
*V* = 2548.89 (11) Å^3^	Needle, colourless
*Z* = 4	0.31 × 0.09 × 0.08 mm

### Data collection

**Table:**

Bruker APEXII CCD diffractometer	6244 independent reflections
Radiation source: fine-focus sealed tube	3307 reflections with *I* > 2σ(*I*)
Graphite monochromator	*R*_int_ = 0.035
φ and ω scans	θ_max_ = 28.3°, θ_min_ = 1.6°
Absorption correction: multi-scan (*SADABS*; Sheldrick, 2008*a*)	*h* = −9→10
*T*_min_ = 0.949, *T*_max_ = 0.987	*k* = −19→21
23203 measured reflections	*l* = −27→25

### Refinement

**Table:**

Refinement on *F*^2^	Secondary atom site location: difference Fourier map
Least-squares matrix: full	Hydrogen site location: inferred from neighbouring sites
*R*[*F*^2^ > 2σ(*F*^2^)] = 0.056	H-atom parameters constrained
*wR*(*F*^2^) = 0.157	*w* = 1/[σ^2^(*F*_o_^2^) + (0.0698*P*)^2^ + 0.2353*P*] where *P* = (*F*_o_^2^ + 2*F*_c_^2^)/3
*S* = 1.01	(Δ/σ)_max_ = 0.001
6244 reflections	Δρ_max_ = 0.19 e Å^−^^3^
276 parameters	Δρ_min_ = −0.14 e Å^−^^3^
0 restraints	Absolute structure: Flack (1983), 2662 Friedel pairs
Primary atom site location: structure-invariant direct methods	Flack parameter: −0.04 (9)

### Special details

**Table:**

Geometry. All e.s.d.'s (except the e.s.d. in the dihedral angle between two l.s. planes) are estimated using the full covariance matrix. The cell e.s.d.'s are taken into account individually in the estimation of e.s.d.'s in distances, angles and torsion angles; correlations between e.s.d.'s in cell parameters are only used when they are defined by crystal symmetry. An approximate (isotropic) treatment of cell e.s.d.'s is used for estimating e.s.d.'s involving l.s. planes.
Refinement. Refinement of *F*^2^ against ALL reflections. The weighted *R*-factor *wR* and goodness of fit *S* are based on *F*^2^, conventional *R*-factors *R* are based on *F*, with *F* set to zero for negative *F*^2^. The threshold expression of *F*^2^ > σ(*F*^2^) is used only for calculating *R*-factors(gt) *etc*. and is not relevant to the choice of reflections for refinement. *R*-factors based on *F*^2^ are statistically about twice as large as those based on *F*, and *R*- factors based on ALL data will be even larger.

### Fractional atomic coordinates and isotropic or equivalent isotropic displacement parameters (Å^2^)

**Table:**

	*x*	*y*	*z*	*U*_iso_*/*U*_eq_	
C1	0.4754 (4)	0.8660 (2)	0.82833 (14)	0.0888 (8)	
H1A	0.5059	0.8075	0.8234	0.107*	
C2	0.3024 (4)	0.8823 (2)	0.79438 (14)	0.0873 (8)	
H2A	0.2679	0.9395	0.8001	0.105*	
H2B	0.2095	0.8476	0.8120	0.105*	
C3	0.3269 (3)	0.86371 (18)	0.72355 (14)	0.0786 (7)	
H3A	0.2150	0.8735	0.7018	0.094*	
H3B	0.3551	0.8057	0.7188	0.094*	
C4	0.4729 (3)	0.91473 (15)	0.69012 (13)	0.0666 (6)	
C5	0.6421 (3)	0.90625 (15)	0.72909 (14)	0.0721 (7)	
C6	0.6235 (4)	0.9177 (2)	0.80067 (14)	0.0901 (9)	
H6A	0.7345	0.9028	0.8213	0.108*	
H6B	0.6005	0.9753	0.8098	0.108*	
C7	0.4179 (4)	1.00624 (17)	0.68853 (16)	0.0969 (9)	
H7A	0.4991	1.0365	0.6620	0.145*	
H7B	0.4205	1.0283	0.7312	0.145*	
H7C	0.2999	1.0110	0.6715	0.145*	
C8	0.7984 (3)	0.89137 (17)	0.70284 (15)	0.0805 (7)	
H8	0.8945	0.8844	0.7303	0.097*	
C9	0.8336 (3)	0.88485 (18)	0.63301 (14)	0.0823 (8)	
H9A	0.8636	0.8282	0.6228	0.099*	
H9B	0.9354	0.9189	0.6224	0.099*	
C10	0.6762 (3)	0.91138 (15)	0.59167 (13)	0.0655 (6)	
H10	0.6733	0.9718	0.5903	0.079*	
C11	0.5021 (3)	0.88085 (15)	0.62200 (13)	0.0653 (6)	
H11	0.5154	0.8211	0.6268	0.078*	
C12	0.3419 (3)	0.8934 (2)	0.57734 (13)	0.0838 (8)	
H12A	0.3119	0.9516	0.5768	0.101*	
H12B	0.2410	0.8640	0.5950	0.101*	
C13	0.3702 (3)	0.86456 (19)	0.50792 (14)	0.0820 (8)	
H13A	0.3805	0.8049	0.5071	0.098*	
H13B	0.2676	0.8798	0.4824	0.098*	
C14	0.5371 (3)	0.90269 (14)	0.47859 (13)	0.0650 (6)	
C15	0.6915 (3)	0.87954 (15)	0.52369 (13)	0.0661 (6)	
H15	0.6880	0.8193	0.5272	0.079*	
C16	0.8581 (3)	0.89897 (19)	0.48523 (13)	0.0841 (8)	
H16A	0.9568	0.8647	0.4989	0.101*	
H16B	0.8911	0.9565	0.4899	0.101*	
C17	0.8063 (3)	0.87974 (18)	0.41608 (15)	0.0838 (8)	
H17A	0.8700	0.8315	0.4010	0.101*	
H17B	0.8355	0.9259	0.3885	0.101*	
C18	0.6013 (3)	0.86341 (15)	0.41517 (13)	0.0683 (7)	
H18	0.5847	0.8037	0.4193	0.082*	
C19	0.5143 (4)	0.88930 (16)	0.35166 (13)	0.0748 (7)	
H19	0.5342	0.9486	0.3463	0.090*	
C20	0.3108 (4)	0.8747 (2)	0.35159 (16)	0.0934 (9)	
H20A	0.2551	0.9125	0.3807	0.140*	
H20B	0.2863	0.8191	0.3648	0.140*	
H20C	0.2650	0.8834	0.3092	0.140*	
C21	0.6022 (4)	0.84559 (17)	0.29503 (14)	0.0828 (8)	
H21A	0.7299	0.8516	0.2989	0.099*	
H21B	0.5753	0.7872	0.2978	0.099*	
C22	0.5466 (4)	0.87628 (18)	0.22971 (15)	0.0924 (9)	
H22A	0.4238	0.8610	0.2222	0.111*	
H22B	0.5535	0.9360	0.2292	0.111*	
C23	0.6578 (5)	0.84295 (18)	0.17692 (17)	0.0880 (9)	
C24	0.6782 (7)	0.8149 (3)	0.06221 (17)	0.169 (2)	
H24A	0.6617	0.8600	0.0331	0.253*	
H24B	0.6373	0.7648	0.0426	0.253*	
H24C	0.8021	0.8098	0.0725	0.253*	
C25	0.2820 (7)	0.8083 (4)	0.1041 (3)	0.206 (3)	
H25A	0.2553	0.7869	0.1459	0.308*	
H25B	0.3244	0.7644	0.0773	0.308*	
H25C	0.1766	0.8316	0.0857	0.308*	
C26	0.5199 (4)	0.99713 (16)	0.47055 (16)	0.0927 (9)	
H26A	0.4993	1.0220	0.5116	0.139*	
H26B	0.4221	1.0092	0.4426	0.139*	
H26C	0.6275	1.0187	0.4525	0.139*	
Cl1	0.45336 (14)	0.88904 (7)	0.91266 (4)	0.1218 (4)	
N1	0.5778 (4)	0.83019 (19)	0.12042 (14)	0.1117 (10)	
O1	0.8116 (3)	0.82332 (15)	0.18387 (12)	0.1129 (7)	
O2	0.4135 (4)	0.86944 (18)	0.10918 (12)	0.1320 (10)	

### Atomic displacement parameters (Å^2^)

**Table:**

	*U*^11^	*U*^22^	*U*^33^	*U*^12^	*U*^13^	*U*^23^
C1	0.0777 (19)	0.097 (2)	0.091 (2)	−0.0030 (17)	−0.0064 (16)	−0.0126 (16)
C2	0.0667 (16)	0.102 (2)	0.093 (2)	−0.0062 (16)	0.0050 (15)	0.0007 (17)
C3	0.0428 (12)	0.1019 (19)	0.091 (2)	−0.0067 (12)	0.0009 (12)	0.0023 (15)
C4	0.0453 (12)	0.0642 (14)	0.0902 (18)	0.0023 (10)	0.0011 (12)	−0.0030 (13)
C5	0.0516 (14)	0.0700 (15)	0.0948 (19)	−0.0081 (11)	−0.0044 (13)	−0.0106 (14)
C6	0.0706 (17)	0.107 (2)	0.092 (2)	−0.0107 (16)	−0.0056 (15)	−0.0163 (17)
C7	0.094 (2)	0.0828 (19)	0.114 (2)	0.0204 (16)	0.0149 (19)	−0.0036 (16)
C8	0.0492 (14)	0.0975 (19)	0.095 (2)	−0.0045 (14)	−0.0098 (13)	−0.0044 (16)
C9	0.0358 (12)	0.0944 (19)	0.117 (2)	0.0002 (12)	−0.0061 (13)	−0.0015 (17)
C10	0.0367 (11)	0.0642 (13)	0.0956 (18)	−0.0018 (10)	0.0029 (11)	−0.0019 (13)
C11	0.0356 (11)	0.0671 (14)	0.0932 (17)	−0.0009 (11)	0.0039 (11)	0.0005 (13)
C12	0.0402 (12)	0.121 (2)	0.0902 (19)	0.0062 (13)	0.0041 (12)	−0.0024 (18)
C13	0.0367 (11)	0.111 (2)	0.098 (2)	−0.0021 (13)	−0.0015 (12)	0.0061 (16)
C14	0.0389 (11)	0.0667 (14)	0.0894 (17)	0.0043 (10)	0.0049 (11)	0.0033 (13)
C15	0.0379 (11)	0.0656 (13)	0.0947 (18)	0.0012 (10)	0.0050 (11)	0.0048 (14)
C16	0.0430 (12)	0.110 (2)	0.099 (2)	−0.0041 (13)	0.0065 (13)	0.0074 (17)
C17	0.0508 (13)	0.0987 (19)	0.102 (2)	0.0067 (14)	0.0164 (14)	0.0180 (17)
C18	0.0510 (13)	0.0613 (14)	0.0927 (18)	0.0052 (10)	0.0045 (13)	0.0092 (13)
C19	0.0675 (15)	0.0658 (15)	0.0912 (19)	0.0138 (13)	0.0064 (14)	0.0027 (14)
C20	0.0644 (16)	0.110 (2)	0.106 (2)	0.0140 (17)	−0.0067 (16)	0.0072 (18)
C21	0.0738 (17)	0.0792 (18)	0.095 (2)	0.0127 (14)	0.0052 (16)	0.0098 (14)
C22	0.092 (2)	0.0893 (19)	0.096 (2)	0.0216 (18)	−0.0097 (18)	−0.0166 (18)
C23	0.089 (2)	0.0821 (19)	0.093 (2)	0.0076 (16)	0.0044 (19)	0.0093 (16)
C24	0.187 (5)	0.238 (5)	0.080 (3)	0.081 (4)	0.019 (3)	0.009 (3)
C25	0.130 (4)	0.230 (6)	0.257 (7)	−0.053 (4)	−0.007 (4)	−0.138 (6)
C26	0.091 (2)	0.0758 (17)	0.112 (2)	0.0209 (15)	−0.0037 (18)	−0.0001 (16)
Cl1	0.1189 (7)	0.1578 (9)	0.0887 (5)	−0.0178 (6)	−0.0027 (5)	−0.0117 (6)
N1	0.111 (2)	0.138 (2)	0.0864 (19)	0.045 (2)	0.0087 (18)	−0.0013 (17)
O1	0.0845 (16)	0.1295 (17)	0.1247 (19)	0.0151 (14)	0.0060 (14)	−0.0026 (15)
O2	0.140 (2)	0.146 (2)	0.1100 (18)	0.051 (2)	−0.0210 (16)	−0.0116 (15)

### Geometric parameters (Å, º)

**Table:**

C1—C2	1.506 (4)	C14—C18	1.547 (4)
C1—C6	1.510 (4)	C15—C16	1.522 (3)
C1—Cl1	1.808 (3)	C15—H15	0.9800
C1—H1A	0.9800	C16—C17	1.528 (4)
C2—C3	1.521 (4)	C16—H16A	0.9700
C2—H2A	0.9700	C16—H16B	0.9700
C2—H2B	0.9700	C17—C18	1.566 (4)
C3—C4	1.543 (4)	C17—H17A	0.9700
C3—H3A	0.9700	C17—H17B	0.9700
C3—H3B	0.9700	C18—C19	1.538 (4)
C4—C5	1.518 (3)	C18—H18	0.9800
C4—C7	1.541 (4)	C19—C21	1.529 (4)
C4—C11	1.541 (4)	C19—C20	1.550 (4)
C5—C8	1.320 (4)	C19—H19	0.9800
C5—C6	1.513 (4)	C20—H20A	0.9600
C6—H6A	0.9700	C20—H20B	0.9600
C6—H6B	0.9700	C20—H20C	0.9600
C7—H7A	0.9600	C21—C22	1.511 (4)
C7—H7B	0.9600	C21—H21A	0.9700
C7—H7C	0.9600	C21—H21B	0.9700
C8—C9	1.486 (4)	C22—C23	1.486 (4)
C8—H8	0.9300	C22—H22A	0.9700
C9—C10	1.527 (3)	C22—H22B	0.9700
C9—H9A	0.9700	C23—O1	1.209 (4)
C9—H9B	0.9700	C23—N1	1.341 (4)
C10—C15	1.515 (4)	C24—N1	1.453 (4)
C10—C11	1.537 (3)	C24—H24A	0.9600
C10—H10	0.9800	C24—H24B	0.9600
C11—C12	1.538 (3)	C24—H24C	0.9600
C11—H11	0.9800	C25—O2	1.405 (5)
C12—C13	1.538 (4)	C25—H25A	0.9600
C12—H12A	0.9700	C25—H25B	0.9600
C12—H12B	0.9700	C25—H25C	0.9600
C13—C14	1.529 (3)	C26—H26A	0.9600
C13—H13A	0.9700	C26—H26B	0.9600
C13—H13B	0.9700	C26—H26C	0.9600
C14—C15	1.542 (3)	N1—O2	1.410 (4)
C14—C26	1.546 (3)		
			
C2—C1—C6	111.1 (2)	C15—C14—C18	100.81 (18)
C2—C1—Cl1	110.1 (2)	C26—C14—C18	109.9 (2)
C6—C1—Cl1	109.0 (2)	C10—C15—C16	119.1 (2)
C2—C1—H1A	108.9	C10—C15—C14	115.59 (19)
C6—C1—H1A	108.9	C16—C15—C14	104.4 (2)
Cl1—C1—H1A	108.9	C10—C15—H15	105.5
C1—C2—C3	108.6 (2)	C16—C15—H15	105.5
C1—C2—H2A	110.0	C14—C15—H15	105.5
C3—C2—H2A	110.0	C15—C16—C17	104.3 (2)
C1—C2—H2B	110.0	C15—C16—H16A	110.9
C3—C2—H2B	110.0	C17—C16—H16A	110.9
H2A—C2—H2B	108.4	C15—C16—H16B	110.9
C2—C3—C4	114.8 (2)	C17—C16—H16B	110.9
C2—C3—H3A	108.6	H16A—C16—H16B	108.9
C4—C3—H3A	108.6	C16—C17—C18	107.3 (2)
C2—C3—H3B	108.6	C16—C17—H17A	110.3
C4—C3—H3B	108.6	C18—C17—H17A	110.3
H3A—C3—H3B	107.5	C16—C17—H17B	110.3
C5—C4—C7	108.9 (2)	C18—C17—H17B	110.3
C5—C4—C11	110.07 (19)	H17A—C17—H17B	108.5
C7—C4—C11	111.2 (2)	C19—C18—C14	119.5 (2)
C5—C4—C3	107.8 (2)	C19—C18—C17	112.6 (2)
C7—C4—C3	109.6 (2)	C14—C18—C17	103.2 (2)
C11—C4—C3	109.2 (2)	C19—C18—H18	106.9
C8—C5—C6	121.0 (2)	C14—C18—H18	106.9
C8—C5—C4	122.8 (3)	C17—C18—H18	106.9
C6—C5—C4	116.2 (2)	C21—C19—C18	110.9 (2)
C1—C6—C5	112.2 (2)	C21—C19—C20	110.9 (2)
C1—C6—H6A	109.2	C18—C19—C20	112.3 (2)
C5—C6—H6A	109.2	C21—C19—H19	107.5
C1—C6—H6B	109.2	C18—C19—H19	107.5
C5—C6—H6B	109.2	C20—C19—H19	107.5
H6A—C6—H6B	107.9	C19—C20—H20A	109.5
C4—C7—H7A	109.5	C19—C20—H20B	109.5
C4—C7—H7B	109.5	H20A—C20—H20B	109.5
H7A—C7—H7B	109.5	C19—C20—H20C	109.5
C4—C7—H7C	109.5	H20A—C20—H20C	109.5
H7A—C7—H7C	109.5	H20B—C20—H20C	109.5
H7B—C7—H7C	109.5	C22—C21—C19	115.2 (2)
C5—C8—C9	125.4 (2)	C22—C21—H21A	108.5
C5—C8—H8	117.3	C19—C21—H21A	108.5
C9—C8—H8	117.3	C22—C21—H21B	108.5
C8—C9—C10	113.4 (2)	C19—C21—H21B	108.5
C8—C9—H9A	108.9	H21A—C21—H21B	107.5
C10—C9—H9A	108.9	C23—C22—C21	113.2 (2)
C8—C9—H9B	108.9	C23—C22—H22A	108.9
C10—C9—H9B	108.9	C21—C22—H22A	108.9
H9A—C9—H9B	107.7	C23—C22—H22B	108.9
C15—C10—C9	112.0 (2)	C21—C22—H22B	108.9
C15—C10—C11	109.96 (19)	H22A—C22—H22B	107.7
C9—C10—C11	109.7 (2)	O1—C23—N1	119.7 (3)
C15—C10—H10	108.4	O1—C23—C22	123.1 (3)
C9—C10—H10	108.4	N1—C23—C22	117.1 (3)
C11—C10—H10	108.4	N1—C24—H24A	109.5
C10—C11—C12	112.1 (2)	N1—C24—H24B	109.5
C10—C11—C4	112.8 (2)	H24A—C24—H24B	109.5
C12—C11—C4	113.62 (19)	N1—C24—H24C	109.5
C10—C11—H11	105.9	H24A—C24—H24C	109.5
C12—C11—H11	105.9	H24B—C24—H24C	109.5
C4—C11—H11	105.9	O2—C25—H25A	109.5
C11—C12—C13	115.00 (19)	O2—C25—H25B	109.5
C11—C12—H12A	108.5	H25A—C25—H25B	109.5
C13—C12—H12A	108.5	O2—C25—H25C	109.5
C11—C12—H12B	108.5	H25A—C25—H25C	109.5
C13—C12—H12B	108.5	H25B—C25—H25C	109.5
H12A—C12—H12B	107.5	C14—C26—H26A	109.5
C14—C13—C12	111.6 (2)	C14—C26—H26B	109.5
C14—C13—H13A	109.3	H26A—C26—H26B	109.5
C12—C13—H13A	109.3	C14—C26—H26C	109.5
C14—C13—H13B	109.3	H26A—C26—H26C	109.5
C12—C13—H13B	109.3	H26B—C26—H26C	109.5
H13A—C13—H13B	108.0	C23—N1—O2	118.1 (3)
C13—C14—C15	106.0 (2)	C23—N1—C24	121.9 (3)
C13—C14—C26	112.0 (2)	O2—N1—C24	113.2 (3)
C15—C14—C26	111.8 (2)	C25—O2—N1	108.1 (3)
C13—C14—C18	115.7 (2)		
